# The perceptions of Lithuanian hunters towards African swine fever using a participatory approach

**DOI:** 10.1186/s12917-022-03509-9

**Published:** 2022-11-14

**Authors:** Evelina Stončiūtė, Alvydas Malakauskas, Franz J. Conraths, Marius Masiulis, Carola Sauter-Louis, Katja Schulz

**Affiliations:** 1 Department of Veterinary Pathobiology, Veterinary Academy, Lithuanian University of Health Sciences, Tilzes 18, 47181 Kaunas, Lithuania; 2 Friedrich-Loeffler-Institut, Federal Research Institute for Animal Health, Institute of Epidemiology, Südufer 10, 17493 Greifswald-Insel Riems, Germany; 3 Emergency Response Division, State Food and Veterinary Service, Siesiku 19, 07170 Vilnius, Lithuania; 4 Dr. L Kriauceliunas Small Animal Clinic, Veterinary Academy, Lithuanian University of Health Sciences, 47181 Kaunas, Lithuania

**Keywords:** African swine fever, Wild boar, Participatory epidemiology, Transdisciplinary, Control measures, Hunting, Lithuania

## Abstract

**Background:**

African swine fever (ASF) has been present in Lithuania since 2014. The disease affects mainly the wild boar population. Thus, hunters play a key role in the performance of disease surveillance and control measures. We used participatory methods to gain insight into the knowledge of hunters and to include their perceptions in the design and the implementation of surveillance and control measures to increase their effectiveness.

**Results:**

The willingness and the interest of hunters to participate was high, but only eight focus group meetings with 33 hunters could be held due to the COVID-19 pandemic. The overall knowledge of Lithuanian hunters regarding ASF, investigated by semi-structured interviews, was sufficient to understand their part in ASF control and surveillance. However, their knowledge did not necessarily lead to an increased acceptance of some ASF control measures, like the targeted hunting of female wild boar. Participating hunters showed a good understanding of the processes of the surveillance system. Their trust in the performance within this system was highest towards the hunters themselves, thus emphasizing the importance of acknowledging their role in the system. Hunters refused measures including the reduction of hunting activities. They feared a complete elimination of the wild boar population, which in turn demonstrates the necessity to increase professional information exchange.

**Conclusions:**

The perceptions of Lithuanian hunters regarding ASF surveillance and control in wild boar resembled those obtained in neighboring countries. It is imperative to communicate the results with decision-makers, to consider the views of hunters, when designing or adapting measures to control ASF in wild boar and to communicate with hunters on these measures and their justification.

**Supplementary Information:**

The online version contains supplementary material available at 10.1186/s12917-022-03509-9.

## Background

African swine fever (ASF) affects suids, like domestic pigs, wild boar and particularly in Africa, where the virus was firstly detected in 1921 [1], several native pig species [2, 3]. While these African native pig species, like warthogs and bush pigs, get infected, they usually do not show any clinical symptoms.

The global interest in the disease has increased, since the virus emerged in Georgia in 2007 [4]. This transcontinental spread led to a continuous dissemination of ASF within Eurasia, which has not come to a halt so far. The disease constitutes a global economic threat for the pig industry and thus requires a high level of awareness of involved stakeholders regarding the characteristics and the epidemiology of the disease. In the current epidemic in Europe, wild boar play an essential role in disease spread and potential virus introduction into domestic pig holdings [5, 6]. Disease control is usually more complex in wildlife than in livestock [7, 8]. The newly characterized wild boar–habitat cycle describes the direct and indirect virus transmission between infected and susceptible wild boar and the environment containing carcasses of wild boar that died from ASF, and it emphasizes the difficulties in ASF control in wild boar [9]. Therefore, the control of diseases with wildlife involvement, such as ASF, requires a transdisciplinary approach. With regard to wild boar, hunters represent the group of persons that normally monitor wild boar populations. By hunting, sampling and actively searching for injured or dead wild boar, they often support surveillance activities and promote disease control [10]. However, it was found that particularly passive surveillance, i.e. the detection, reporting and sampling of wild boar found dead, is very unpopular within the hunters’ community [10]. Despite the reluctance of hunters to support these aspects, passive surveillance remains one of the most important components for ASF surveillance and control [5, 11, 12]. Enhanced searches for wild boar carcasses are of utmost importance for detecting disease introduction early. Through the removal or safe disposal of infected wild boar carcasses, further transmission may be prevented and the disease contained. To ensure effective and successful passive surveillance, the willingness of hunters to participate is essential. In participatory studies conducted with Estonian and Latvian hunters, motivational options and favored ASF control measures were investigated [13, 14]. There was relative agreement in the two countries regarding preferred control measures and the options that may increase the motivation of hunters to support passive surveillance [15].

However, the epidemiological course of ASF in Lithuania seemed to differ from the one in Estonia and Latvia [16]. The containment of the ASF virus was slower, potentially due to an inadequate implementation of biosecurity measures at hunting ground level. ASF virus circulation in the wild boar population has been continuously reported and disease elimination does currently not seem to be within reach. To implement ASF control measures more effectively, transdisciplinary cooperation is needed, involving hunters to support ASF control even more. Evaluating the current level of knowledge and willingness to support the implemented measures, a questionnaire addressing the knowledge of hunters about ASF and their perceptions towards ASF control and passive surveillance was administered [17]. Although the results from the questionnaire-based surveys resembled the results of participatory studies conducted in Estonia and Latvia, deeper insights into the motivations behind the hunters’ statements could not be obtained by this approach, since mostly closed questions were asked. Consequently, a participatory study was designed based on the questionnaire results. The aim was to use the advantages of participatory methods to investigate the knowledge of hunters regarding ASF in wild boar in more detail and to study the perceptions of hunters towards control measures and their trust in ASF surveillance and control. The results of the study may be used to close knowledge gaps in hunting communities and thus to increase the acceptability of necessary control measures. Specific measures may be designed or adapted taking the perceptions of hunters into account. However, another main objective of the study was to improve transdisciplinary communication and cooperation in order to promote the common goal of successful ASF control, which can only be achieved through collective action.

## Results

### Participating hunters and focus group meetings

**Fig. 1:**
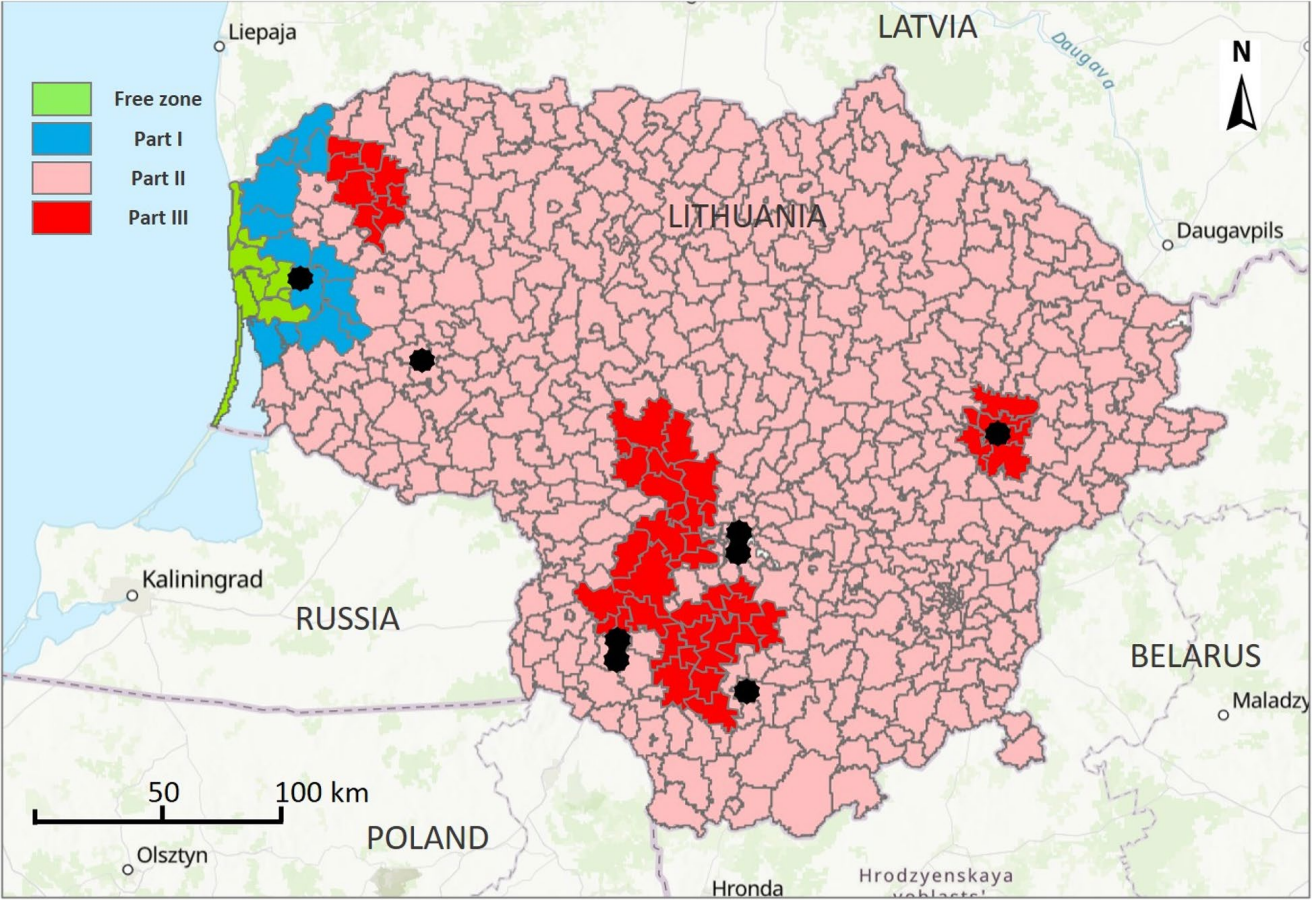
Locations of focus group meetings (black points) and actual distribution of different ASF restricted zones during August – October in 2020 in Lithuania

In total, eight meetings were conducted. Hunters (*n* = 33) from six out of ten Lithuanian counties were involved in the study (Table 1). One meeting took place in an area regionalized according to “Commission Implementing Decision 2014/709/EU of 9 October 2014 concerning animal health control measures relating to African swine fever in certain Member States and repealing Implementing Decision 2014/178/EU” in the restricted zone I (Part I) and another one in the restricted zone III (Part III), while the remaining meetings took place in areas categorized as restricted zone II (Part II) (Fig. 1). One group consisted of one woman and three men, another group exclusively of women and six only of men (Table 1). The estimated age of the participants ranged from 20 to 80 years. The estimated average age was approximately 50 years. The duration of the meetings varied from 1 h 15 min to 2 h 04 min.

**Table 1:** Locations of focus group meetings and number of participating hunters in these meetings in 2020 in Lithuania

County	No. of participating hunters	Gender composition within the groups		ASF restricted zone
		**Male**	**Female**	
Alytaus	5	5	0	Part II
Marijampolės	4	4	0	Part II
Marijampolės	4	3	1	Part II
Kauno	3	3	0	Part II
Kauno	4	0	4	Part II
Tauragės	4	4	0	Part II
Klaipėdos	5	5	0	Part I
Utenos	4	4	0	Part III

### 1. Part: knowledge assessment

The results of the knowledge assessment are divided in the four subtopics discussed with the participating hunters. The four code groups, their respective subgroups and the numbers of mentioning can be found in supplementary file, Table 1.

#### a. Known ASF control measures

Hunters were asked questions on their knowledge on ASF control measures. Removal of carcasses (mentioned five times) and ban of supplementary feeding (mentioned four times) were ASF control measures most frequently listed by the hunters during discussions. Both measures were heavily criticized by the participants. As mentioned earlier, carcass removal by burying was considered as a problematic way of disposal due to the lack of functional digging tools to complete this task sufficiently under field conditions. Ban of supplementary feeding was assumed to increase the viral spread further, because it forces wild boar to move longer distances in search of food.

Participants mentioned wild boar population control, physical barriers, and biosecurity implementation as ASF control measures. All these measures were considered as necessary, although a few hunters mentioned that some of the biosecurity requirements (like mandatory disinfection of apparel before and after hunting) are too laborious. Support for a ban of driven hunts (particularly if conducted with dogs) was expressed once. Supporting participants claimed that it might have been advantageous, if this ban had been implemented earlier, since driven hunts potentially increase the movement of wild boar, which may lead to ASF spread.

#### b. Consequences of ASF persistence in the Lithuanian wild boar population

Hunters discussed that ASF persistence had the biggest impact on wild boar population density. They mentioned this possible consequence nine times. Hunters expressed concern that the expected decrease in population density might reduce their chance to hunt wild boar and thus their pleasure in hunting. Participants expressed that they indeed noticed a significant decrease of the wild boar population, after ASF had been introduced into Lithuania. Since some hunters consider wild boar as the main game species, some of them lost their motivation to go hunting, now perceiving their hobby rather as a duty than a joyful activity. The thought that the persistence of ASF reduces the joy in hunting as a hobby or leisure activity was mentioned eight times during discussions. Hunters added that the current procedure of testing wild boar samples for ASF was inconvenient, not only because of the time, they have to wait for the results, but also due to a in their view poorly organized coordination of sample transportation. Hunters admitted that this reduced their willingness to participate in sampling overall. Inconvenient ASF sampling was mentioned four times during discussions with the hunters.

Hunters added that the implementation of some measures caused financial losses to them, which were not compensated by the government (mentioned four times). As an example, participants argued that they had to pay for disinfectants required to dispose the carcasses safely. Some participants also mentioned own costs for equipment used for the refrigeration of hunted wild boar while waiting for ASF test results.

Participants stated that the persistence of ASF had a significant economic impact on pig keepers (mentioned four times during discussions). Some hunters argued, that ASF control should be the pig keeper’s responsibility in the first place.

#### c. Potential routes of virus introduction into the Lithuanian wild boar population

Various fomites imported from other affected countries were mostly mentioned by hunters as possible reasons for ASF introduction. Farm animal feed, imported wood and/or food waste discarded by passing drivers from neighboring countries like Russia or Belarus were listed nine times as a potential source of virus introduction. Wild boar movement from affected countries was mentioned six times as a potential route of virus introduction. Hunters explained that sick, migrating wild boar from affected neighboring countries might have contact with Lithuanian wild boar, which may lead to the spread of ASF within the national wild boar population. Two times hunters mentioned domestic pig holdings as a possible route of introduction, explaining that infected pigs, purchased for breeding, might have been imported from countries affected by ASF.

#### d. Transmission pathways of the virus

Hunters most frequently expressed the view that ASF virus is transmitted through people movement (mentioned 12 times), migrating sick animals (mentioned 11 times), and a general lack of biosecurity (mentioned 10 times). The participants explained that people might carry the virus on their clothes and footwear. Hunters mentioned that ASF virus might be distributed by berry- or mushroom pickers, loggers and other forest visitors that are not obliged to comply with biosecurity measures. Some participants speculated that the human factor is the most effective way of spreading the virus and that it is even more effective than direct contact between wild boar.

In two groups it was mentioned that the previous policy of paying a financial reward to persons who found and reported a wild boar carcass might have increased the movements of people in the forests affected by ASF and that this could have supported the further distribution of ASF.

Eight times hunters mentioned that the movement of infected wild boar is closely related to the ban of supplementary feeding. They explained that the ban of additional feeding could result in an increased home range of wild boar searching food. This change in behavior could support the spread of ASF.

Four times participants also listed wild boar carcasses as a possible source for ASF spread. Hunters completely agreed that carcasses should be removed, but many of them believed that burying carcasses is a wrong way of disposal. They argued that it is often difficult to bury the body deep enough to avoid excavation by scavengers. Therefore, they feel often forced to move the carcass to a place, where they can safely bury it, thus spreading ASF on the way. Hunters in one of the discussion group believed that the ASF virus may stay infectious underground for a longer period and that the carcasses should be collected and burned.

Contaminated trash and food waste were mentioned three times as a possible way to transmit ASF. Hunters explained, that people leave food and meat waste near the woods and that wild boar as omnivores may consume contaminated products and might thus get infected with ASF. Participating hunters regarded contact between wild boar as a way of transmitting ASF. This option was mentioned twice during discussions.

### 2. Part: evaluation of the acceptability

The acceptability of measures was studied in the focus discussion groups. First, the members of the groups were asked to describe the ASF control network in Lithuania. In relation diagrams drawn during the meetings, 14 different stakeholders were mentioned (Table 2). The most frequently named stakeholders were the State Food and Veterinary Service of the Republic of Lithuania (SFVS) (mentioned 13 times during discussions in all eight groups) and hunters (mentioned eight times in all eight discussion groups). Forest visitors were named by five groups and three groups listed hunters and pig keepers as part of the ASF control network.

**Table 2:** Stakeholders involved in the control of ASF in wild boar named by the hunters in the individual focus group meetings in Lithuania

Stakeholder	No. of focus groups that mentioned the stakeholder
State Food and Veterinary Service	8
Hunters	8
Ministry of the Environment	7
Other forest visitors	5
Pig keepers, farmers	3
Scientific institutions	2
Government	2
Hunting associations	2
Ministry of Agriculture	2
Non-governmental institutions, Social initiatives	2
Private veterinarians	1
European Union (EU)	1
Foresters	1
Parliament	1

**Fig. 2:**
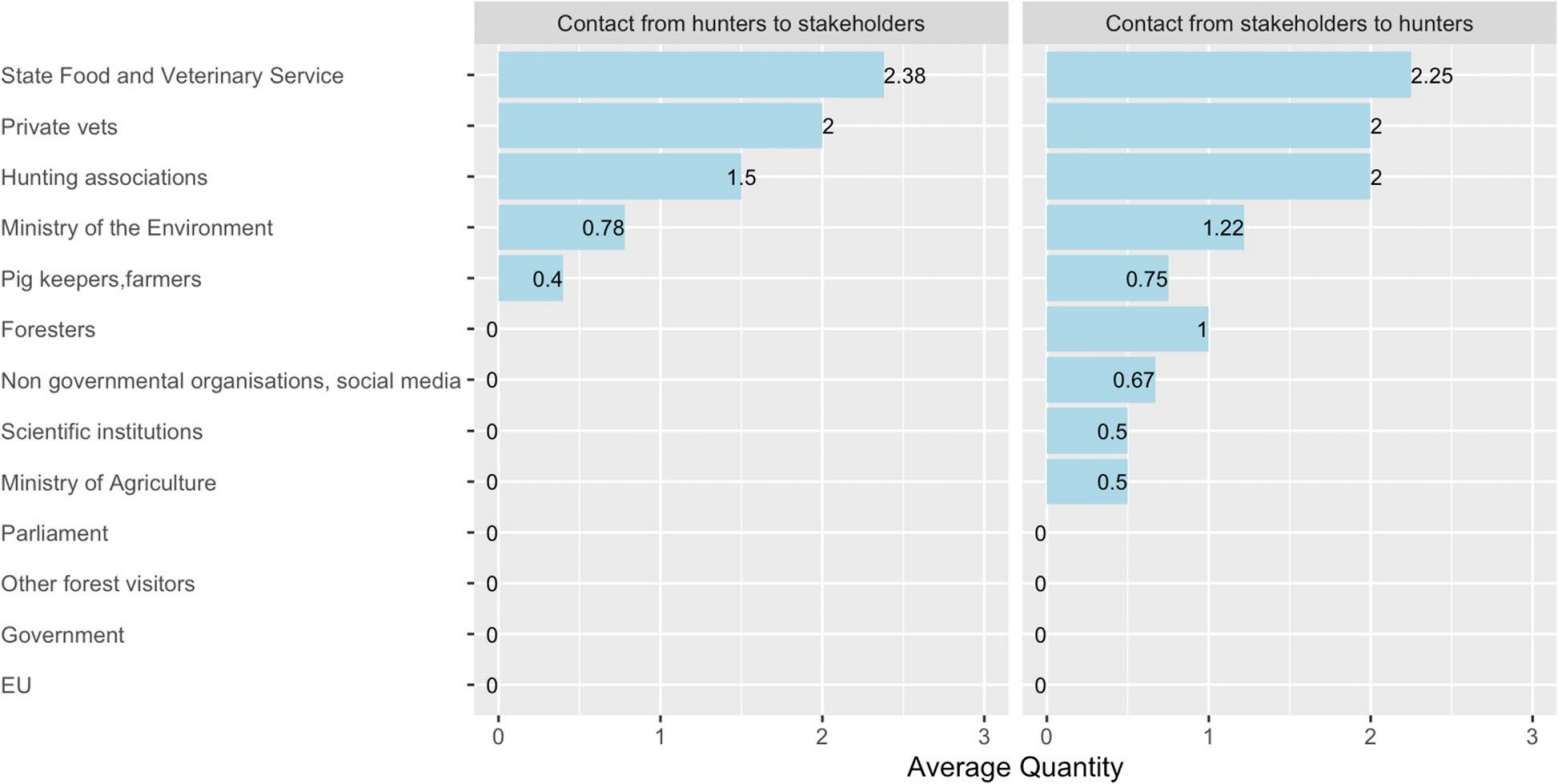
Frequency of contact from hunters to stakeholders involved in ASF control and the frequency of contact from stakeholders to hunters (0 – no contact; 1 – rare contact; 2 – regular contact; 3 – daily contact)

The most frequently named contact was that between the SFVS and the hunters, with more frequent contacts from the hunters to the SFVS than vice versa. Hunters also reported frequent contact to private veterinarians and to various hunting organizations. Contacts with governmental institutions like Ministry of the Environment, Ministry of Agriculture as well as contact with pig keepers, foresters, scientific and non-governmental institutions were regarded as rare. Several stakeholders, like foresters, non-governmental organizations, scientific institutions and Ministry of Agriculture only contacted hunters, whereas hunters did not contact these stakeholders (Fig. 2).

The quality of the contacts within the ASF network correlated with the quantity of the contacts. Thus, hunters were most satisfied with the contact to private veterinarians followed by SFVS and hunting associations. Despite most participants’ general satisfaction with their contacts to the SFVS, some of them perceived a lack of involvement of hunters in ASF control and cooperation between the SFVS and the hunters.

Although the hunting associations were considered as the most important link between hunters and the governmental institutions, some participants mentioned crucial information (e.g., government issued laws and orders) was usually poorly transmitted to hunters despite regular contacts.

**Fig. 3:**
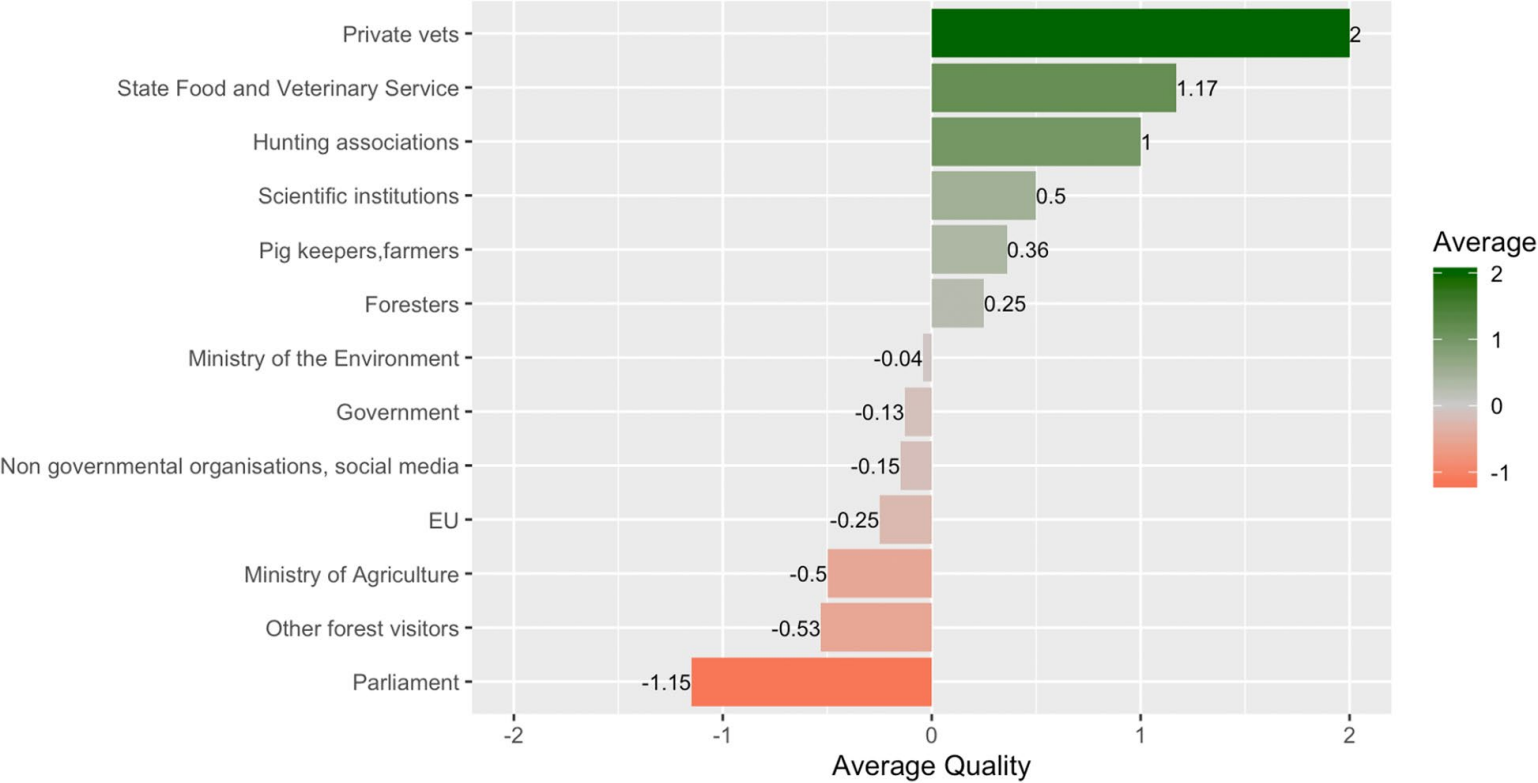
Stakeholders listed by hunters in the relation diagram and the calculated average quality of the contacts between them and the hunters

The rare contact to scientific institutions was regarded as slightly positive. Participants explained that this stakeholder is only in contact with them regarding sample collection for research or in case of training offers on infectious diseases, which was highly appreciated by the hunters. Some participants stated that they miss scientifically based decisions in ASF control and that the involvement of scientific institutions in ASF surveillance and control could be more prominent. Rare or non-existing contacts with stakeholders like Ministry of Agriculture, Parliament and the EU were judged as negative. The main argument was that the only role of these institutions in the control of ASF was to set up regulations, which were regarded as often too theoretic, sometimes hardly applicable and far away from reality (Fig. 3).

**Fig. 4:**
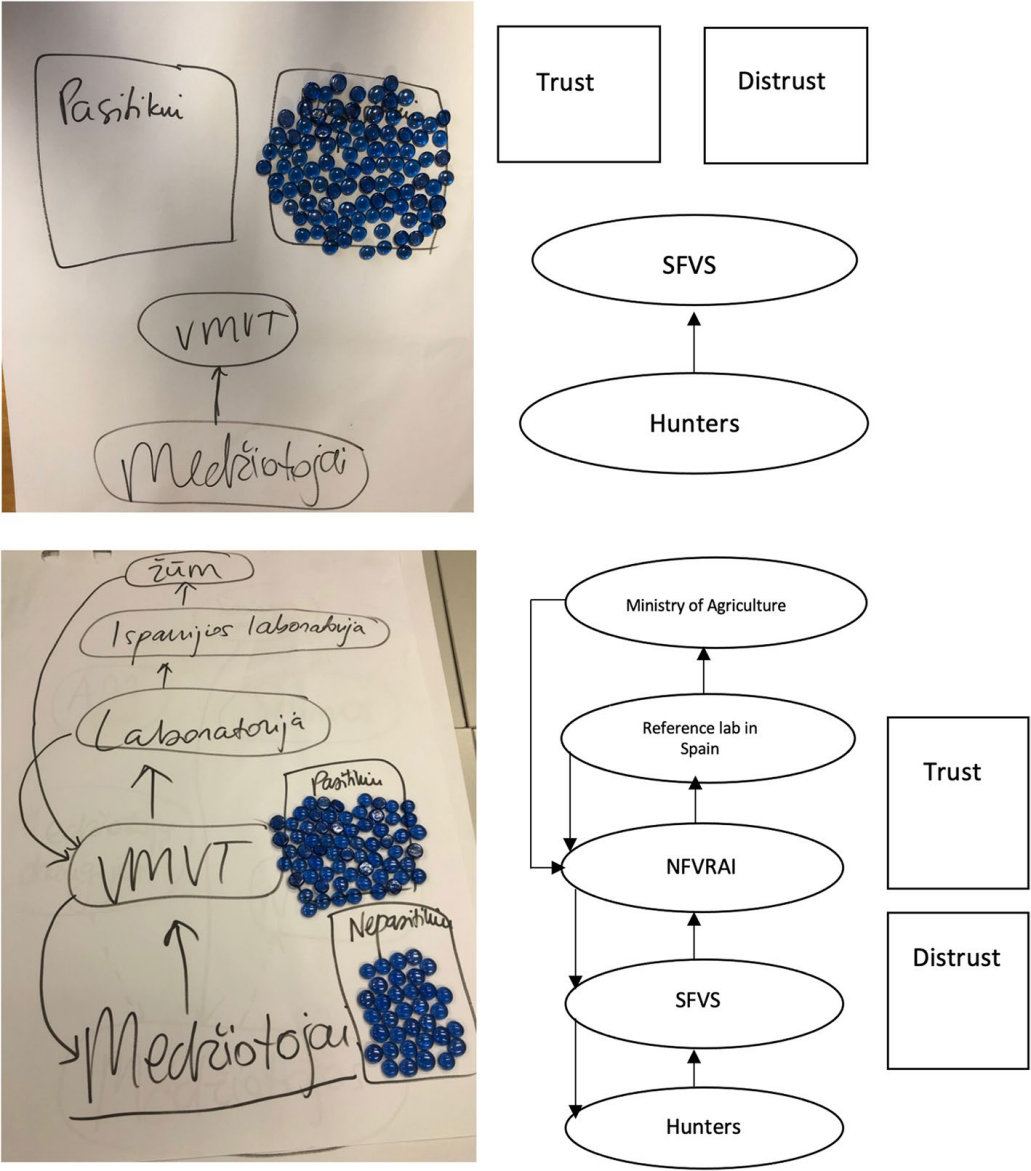
Information flow in the Lithuanian ASF surveillance and control system as perceived in two representative focus group discussions (The upper schematic example illustrates a basic understanding of the ASF surveillance and control system provided by one of the participating groups, the lower example demonstrates a more detailed overview of the ASF surveillance and control system described by other participants)

The majority of hunters was aware of the basic structure of the ASF control system in Lithuania. The participants could not only list the stakeholders, whom they directly contact on a daily basis, but also named further stakeholders and governmental structures responsible for various aspects of ASF control (Fig. 4, lower example. The group came from an ASF restricted zone II, Kaunas county). One group of hunters was only able to list the direct stakeholder, SFVS, to whom they submitted the collected wild boar samples This group could not name any other governmental structures involved in the ASF control system (Fig. 4, upper example. The group came from an ASF restricted zone II, Alytus county). The stakeholders listed most frequently in the flow diagrams by all participating hunters were: SFVS, National Food and Veterinary Risk Assessment Institute and Ministry of Agriculture.

The average trust (50.25%) of hunters in the ASF surveillance system in Lithuania was almost equal to the distrust (49.75%) in the system. Hunters explained that, in their opinion, individual stakeholders do their job efficiently, but the system as a whole does not perform as well as it should. Participants argued that some implemented measures lack reasoning, that there was too much bureaucracy and that government officials were not acquainted with the situation in the field. Hunters expressed disappointment that decision-makers failed to follow successful examples from other countries.

Participants trusted in the hunters most regarding effective implementation of ASF control measures. They explained that hunters were motivated to eliminate the disease from the wild boar population and that they tried to support the implemented measures as much as they could (Fig. 4). The SFVS was regarded as the second-most trusted stakeholder in the ASF control system. Hunters explained that SFVS only executed measures that had been determined by the higher authorities. However, in the view of the hunters, the SFVS does its job efficiently. Some participants explained that, in the beginning of the ASF epidemic in Lithuania, the SFVS had been very fast in punishing hunters, if they had made mistakes, rather than aiding. This may have damaged the relationship with this stakeholder (supplementary file, Fig. 1).

Participants perceived the National Food and Veterinary Risk Assessment Institute (NFVRAI) as the third-most trusted institution in the system. They had no complaints about the reliability of ASF test results and regarded the work of the institute as efficient and important. Regional branches of the SFVS, serving as the local governmental offices, were regarded as trusted stakeholders helping hunters with most of their common issues. Still, some of the participants negatively mentioned logistical problems leading to sample delivery to regional SFVS outside regular working hours, which causes additional inconveniences for participating hunters.

The Ministry of the Environment and EU institutions were the least-trusted stakeholders. Hunters believed that these institutions are responsible for the order of flawed ASF control measures implemented in Lithuania. According to the participants, the Ministry of the Environment failed to communicate appropriately with the hunters. The ministry was perceived as being unwilling to provide any education or training to the hunters. Furthermore, the direct contact of hunters with staff of the institution was perceived as unpleasant (supplementary file, Fig. 1).

### 3. Part: evaluation of ASF control measures

#### (1) Including additional forces like army and police in ASF control (for shooting wild boar and/or carcass search)

Most participants considered the inclusion of additional forces for carcass search or wild boar shooting as an ineffective measure to control ASF. Hunters believed that this measure might have been effective in the beginning of the ASF epidemic in Lithuania, but that it was of no use in the current situation. Participants argued that additional people in the woods might bring more chaos and increase the risk of further virus spread. In addition, hunters noted that neither the army nor the police have the necessary skills to hunt wild boar. Therefore, their involvement could result in more injured animals. The cost for equipping and training soldiers would be too high. Although hunters did not support the idea of any additional forces contributing to ASF control, some participants considered their involvement as potentially beneficial in searching or burying carcasses and controlling the movement of people in the woods in areas highly affected by ASF.

#### (2) Selective hunting of female wild boar

One of the groups agreed that selective hunting of female wild boar might be effective to control ASF. However, the majority of participating hunters explained that they would not apply it in the field, as they considered it as inhumane. They agreed, however, that selective hunting of female wild boar might be an efficient way to control the wild boar population. Participants mentioned that they might be less reluctant to support this measure, if there was scientific evidence that diseased female wild boar could transmit the virus to their offspring. Due to concern that the wild boar population could be totally wiped out in Lithuania by ASF and ASF-related control measures, some participants claimed to be more supportive towards this measure in case of an official limit on the number of female wild boar that would be allowed to be hunted. Introducing an upper limit, depending on the local population density, may thus help to keep the wild boar population smaller, but healthy. Finally, participants argued that selective hunting would almost be impossible without the right hunting tools, e.g., night-vision device, even if the hunters were willing to apply them. Hunters explained that with the help of these tools, they could selectively hunt older females moving long distances, potentially together with their pack and consequently spreading ASF further.

#### (3) Ban of supplementary feeding

Most participants agreed that contrary to the widespread opinion, additional feeding was a measure that helps to control ASF as it may reduce wild boar movement. Some participants argued that without supplementary feeding, wild boar would enter the neighboring farmland, destroy crops, thus causing trouble to farmers and hunters, and spread ASF further. Participants added that the existence of a defined feeding spot could help to reduce the population size and increase the number of hunted wild boar. In one group, it was mentioned that the effectiveness of the feeding ban depends on the amount of feed given to the animals. The ban could be justified, if too much feeding is supplied, thus increasing the number of direct wild boar contact and ASF transmission. If less feed was used, this may not increase the risk of disease spread. One group of hunters suggested that the feeding ban is an effective measure and that supplementary feeding would only be beneficial in areas with a high ASF prevalence. It might help to keep the wild boar local and avoid movements to other hunting grounds.

#### (4) Ban of driven hunting

The majority of participants did not regard a ban of driven hunts as effective. Hunters thought that it was based on the wrong assumption that wild boar move to other hunting grounds, when they are under hunting pressure. Hunters explained that wild boar are territorial animals and that they swiftly return to a territory, from which they were forced to leave. Participants criticized the lack of evidence regarding the effectiveness of this measure. They also believed that the ban of driven hunts contradicts the current national objective to reduce wild boar populations efficiently, since usually the largest number of wild boar is shot in driven hunts. In addition, they argued that more wild boar carcasses are found during driven hunts. The hunters frequently mentioned that they were not willing to support the ban on driven hunts, because they represent enjoyable social events, at which they can meet other members of the hunting community.

Hunters in three different discussion groups agreed with the ban on driven hunts because of the potentially increased movements of wild boar. In one group, a few hunters argued, that driven hunts with dogs should only be banned, as dogs chase wild boar farther than people. Some hunters added that this measure might be effective, if it was implemented in areas affected by ASF more severely.

#### (5) Ban of individual hunting

None of the groups agreed with the ban of individual hunting. Participants explained that they had no opportunity to hunt wild boar selectively, if individual hunting was banned. The hunters argued individual hunting was the best and most efficient way to hunt. In their view, the animals were usually shot dead according to the rules and less frequently only wounded. Wild boar shot by individual hunting can be collected in the safest way. Participants added that hunting in raised hides makes it easier for them to comply with the required biosecurity measures. Instead of walking through the forests and potentially spreading the disease, while following the prey, they can conveniently use their vehicle to transport hunted animals, if they are shot near the hunting tower. The participants could not suggest any advantage in a ban of individual hunting for controlling ASF in any situation.

## Discussion

We employed a participatory approach to study the knowledge of Lithuanian hunters towards ASF and to investigate their perceptions regarding ASF surveillance and control. Hunters are paramount in wild boar population management and thus play a major role in the surveillance of ASF in wild boar. In recent studies, the use of participatory methods has demonstrated their potential to increase inter- and transdisciplinary communication, to improve mutual understanding and thus to support successful disease control [18–21]. Participatory studies focusing on ASF control have so far have mainly looked at the attitudes of farmers [22–25]. However, in Estonia and Latvia, participatory methods were used to evaluate the perceptions of hunters regarding ASF surveillance and control in wild boar [13, 14]. In Lithuania, a conventional online questionnaire with similar questions was administered to hunters [17]. The questionnaire, which included mainly closed questions, revealed a comparable picture of hunters' opinions regarding ASF to the results of the studies in Estonia and Latvia. However, to take advantage of participatory methods and to gain a deeper insight into Lithuanian hunters' perceptions of ASF surveillance and control, the present participatory study was conducted. In contrast to the studies in the other two Baltic countries, the design of the participatory study was based on the results that had been obtained in the survey based on a questionnaire. This allowed designing the research questions in a more detailed and targeted way. The character of a semi-structured interview and of focus group discussions implies open end questions, which, in contrast to a conventional questionnaire with closed questions, offers the possibility of elucidating themes that the researcher had not even considered in the planning of the study.

In total, eight meetings were conducted, in which 33 hunters participated. This number of participants was lower than in similar studies [13, 14]. However, in the study of Schulz et al. [10], which used similar methods, only 28 hunters participated. Saturation was still reached. Originally, more hunters registered for the study presented here, but due to the COVID-19 pandemic and its consequent restrictions, only eight meetings could be held. Nevertheless, the benefit of further meetings seems to be negligible, since a high agreement between the groups was observed. It is to be assumed that also in the present study, saturation was reached [26]. We aimed to involve hunters from different ASF restricted zones [27] to include various experiences about implemented ASF surveillance and control measures. However, the statements of hunters from the different groups and regions were similar. Results did not differ between different gender groups. The only notable difference was that the women were less elaborate on their opinions and added less additional information.

While the benefits of a participatory approach are undisputed, their application is often challenging. The moderator of the meetings received a basic short training in fundamental principles of participatory epidemiology. However, it is known that good skills in communication and some training are required to ensure that power imbalances do not hamper a balanced and diverse discussion [28, 29]. Thus, biased results yielding from unbalanced group dynamics cannot be excluded completely. However, due to the overlaps in content between the different groups, it can be assumed that any potential bias did not affect the results in a crucial way. Both, the moderator and the observer came from academia, which probably supported the candor of the hunters. However, the presence of university staff might also have caused uncertainty among the hunters and thus influenced the results.

In contrast to the studies from Estonia and Latvia [13, 14], where no semi-structured interviews were conducted, knowledge of the hunters on national ASF surveillance and control system with the focus on wild boar was assessed using a semi-structured interview. Consequently, a large amount of information was gathered including contributions to the discussions, which were partly unexpected. The statement that financial incentives for the detection of wild boar carcasses could indirectly have supported the virus spread was interesting and should certainly be considered, when planning such rewards. In the study of Jori et al. [30], experts expressed similar concerns. Generally, the results of the semi-structured interview indicated that Lithuanian hunters have a relatively good basic understanding of ASF in wild boar.

The investigation of hunters’ knowledge on ASF transmission and control was done under the assumption that a sound knowledge about ASF goes hand in hand with an increased willingness to support disease surveillance and control measures. However, despite the apparently good knowledge of Lithuanian hunters about ASF, which was demonstrated in the results of the present study and in that of Stončiūtė et al. [17], some of their statements suggested that our assumption was not entirely correct. Although hunters partly agreed that some of the discussed ASF control measures might be effective, they listed several arguments, which speak against active support by the hunters. Also, the ban of supplementary feeding, which is scientifically discussed as an effective measure to prevent the direct contact of different wild boar packs and may support population reduction, particularly in harsh winters [6, 31], was rejected by the hunters. On the contrary, it was even considered counterproductive, as it might lead to an increased movement of wild boar. These findings were similar to those from Estonia, where hunters denied any inhibiting effect of the ban of supplementary feeding on the spread of ASF [13].

The fear that some of the ASF control measures (like hunting female wild boar) could wipe out the entire wild boar population is evidence of inadequate education of hunters and their great desire to have a sufficiently large number of wild boar to hunt. The concerns of the participants that ASF could reduce the wild boar population to a level, at which wild boar hunting would be impossible, could however be used to adapt the motivation of hunters to actively support ASF control. The need for more professional information exchange could also be seen by the fact that hunters stated the wish that stakeholders should learn from countries that have managed to eliminate ASF successfully. However, due to the different epidemiological settings in the different countries, Lithuania and its requirements for disease control cannot be compared with the ones in the Czech Republic or Belgium [32], two previously affected countries that were successful in eliminating ASF from wild boar populations. This should be explained to and discussed with the hunters.

The frequent and positively perceived contact of the hunters with the SFVS and the other relations within the hunting network resembled the results obtained in Estonia and Latvia [13, 14]. Hunters expressed the wish to get more scientific information, which should not be ignored but taken as an opportunity to improve transdisciplinary contacts.

Under the assumption that trust and satisfaction in a defined network increase the acceptability of the operations within the system, a flow diagram was used. Similar to other studies, the trust in hunters regarding their performance (hunting, removing wild boar carcasses from the environment and sample collection) within the system was high [13, 14]. Thus, an adequate recognition of their contribution to ASF surveillance and control in wild boar is necessary to ensure good cooperation.

The control measures that were presented to the hunters were chosen by the authors depending on the results of the questionnaire. Scientifically, these measures are still regarded as potentially effective [5, 6], thus the aim was to investigate reasons for their unpopularity found in the questionnaire study of Stončiūtė et al. [17] in the discussions and to evaluate possibilities to increase the acceptance of these measures by hunters. Hunters agreed with the results from the questionnaire and confirmed the unpopularity of the chosen ASF control measures. Thus, the perceptions of the hunters regarding the presented control measures did not differ much from those recorded in recent studies [13, 14, 17]. However, Lithuanian hunters did not generally reject the involvement of the army. In contrast to Estonia [13] and Latvia [14], the arguments against the targeted hunting of female wild boar were dominated by the fear that the wild boar population could be eliminated, indicating again the urgent need for active information exchange. As in previous participatory studies involving hunters [13, 14, 17], hunting bans were very unpopular. Although a few hunters agreed to the potential effectiveness of the ban of driven hunts to avoid further virus spread, the majority was very clear in declining these measures. These statements again suggest that hunters may lack information.

The study results should be shared with decision-makers. In Lithuania, the disease is still present and it does not seem that elimination of the virus is imminent [16]. Therefore, actively seeking contact to the hunting community seems necessary to identify starting points for a more fruitful cooperation between the relevant groups of persons and successful disease surveillance and control. Despite the already gained knowledge, further participatory studies in different countries and different epidemiological ASF settings should be performed to increase the chances of effective ASF control in wild boar. In future studies, more attention should be paid to sufficient training of the study personnel (in particular moderators and observers) in methods adopted from social sciences. Also, the spectrum of participating hunters should be exceeded to ensure that the entire opinion of the hunting community is represented.

## Conclusions

This study is part of a series in evaluating the perceptions of hunters with regard to ASF control [13, 14, 17], thus acknowledging the key role of hunters in ASF surveillance and control. The results are comparable within Lithuania, but also between the three Baltic States. Particularly the rejection of certain ASF control measures was expressed by the majority of hunters. Still, it was important to investigate the perceptions of Lithuanian hunters and to add this knowledge about the opinions of hunters regarding ASF in wild boar. Transdisciplinary communication has to be improved and consolidated. Cooperation needs to be conducted on an equal footing. The objections of hunters must be perceived by decision-makers and solutions must be found together. The next step in increasing the chances of success in disease control by using participatory epidemiology may be real participation, which means not only asking for opinions, but also to allow joint determination of goals and joint decision making together with the hunters. If this is not achieved, there is the danger that ASF will remain endemic in wild boar in many European countries, thus continuously threatening the domestic pig industry. This disease can only be successfully and sustainably combated together.

## Methods

All methods used in the present study were carried out in accordance with relevant guidelines and regulations.

### Recruitment of participants

Between August and September 2020, hunters were invited to participate voluntarily in focus group meetings by placing an invitation on the most popular Lithuanian hunting website (www.miske.lt) and sending it to hunting associations in Lithuania. The invitation contained a link to an online registration form (https://forms.office.com) and all willing participants registered by providing their contact information, age, gender, hunting area and preferred meeting place. During the registration, hunters were informed about the aims of the study and of anonymous publication of the results and by filling in the registration forms, the hunters gave their written informed consent to the anonymous use and publication of the gathered data. Also, before each meeting, hunters were again orally informed about the anonymous publication of the data and all participating hunters provided their oral informed consent personally. Except for the willingness of the hunters to participate, there were no other inclusion or exclusion criteria to participate in the study. When organizing the meetings, the aim was to preferably include participants from different parts of Lithuania and from different epidemiological ASF restricted zones (Fig. 1). Hunters were invited and the meeting dates fixed by phone depending on the availability of the majority of hunters, with the aim of including three to seven hunters per group discussion, and holding the meetings within a reasonable period after the invitation. Some planned meetings had been cancelled and the participants of others re-arranged due to the SARS-CoV-2 situation.

### Focus group meetings

In each focus group meeting, three to five hunters participated. The meetings were conducted in October 2020. All discussions were facilitated by the same moderator and monitored by the same observer. Both, the moderator and the observer had previously participated in a one-day training course to become familiar with basic principles of participatory methods. The moderator and the observer were members of the Department of Veterinary Pathobiology of the Veterinary Academy of the Lithuanian University of Health Sciences and native speakers of the Lithuanian language. During the meetings, the role of the observer was solely to observe the meetings without intervening at any time. The moderator facilitated the meetings and explained the implemented tools. The participants themselves used the tools with the support of the moderator.

The meetings were divided into three parts to evaluate three different aspects related to ASF in wild boar. By conducting a semi-structured interview, we aimed to assess hunters’ general knowledge about ASF in the first part of the meeting. To evaluate the hunters’ acceptability of the ASF surveillance and control system in the second part, diagrams, visualization and ranking tools adapted from Schulz et al. [10] and Calba et al. [33] were used. In the last part, ASF control measures that were previously considered as ineffective by Lithuanian hunters in a questionnaire survey [17] were evaluated by the participating hunters within the focus group discussions. All discussions in the semi-structured interviews, while implementing the participatory tools and during the focus group discussions were analyzed descriptively using “ATLAS.ti” software (Version 8.4.3). The conversations were recorded using a “SONY ICD-PX370” dictation device. The meetings were held in Lithuanian language. Subsequently, the transcripts of the recordings were translated into English by three qualified members of the Department of Veterinary Pathobiology.

The discussions were coded into code groups and subgroups. Code groups were based on the general broad topics analyzed: “Consequences”, “Control measures”, “Virus introduction” and “Virus transmission”. Each code group was subdivided into subgroups according to the main ideas that were expressed regarding a specific topic. For example, code group “Virus introduction” was subdivided into three subgroups based on the ideas, participants had raised: “Migration of wild boar from neighboring countries”, “Pigs breeding farms” and “Various fomites import from affected countries” (supplementary file, Table 1). All codes of subgroups were associated with specific participants’ quotes in the meetings transcriptions and the frequency of mentioning specific codes in any of the three parts of a meeting was counted. All parts of the discussions were categorized and coded in the same manner (supplementary file, Table 1).

#### 1. Part: knowledge assessment

A semi-structured interview was conducted to evaluate the general knowledge of hunters about ASF. Prior to the meetings, a checklist was prepared to ensure a relatively uniform and complete coverage of the topics of interest. The following questions were discussed:

- Known ASF control measures.
- Consequences of ASF persistence in the Lithuanian wild boar population.
- Potential routes of virus introduction into the Lithuanian wild boar population.
- Transmission pathways of the virus.

All questions were formulated as open-ended questions. To ensure a correct understanding and interpretation of the statements of the participants, regularly probing was performed by the moderator.

#### 2. Part: evaluation of the acceptability

A relation diagram was used to assess hunters’ relationships to and their satisfaction with other stakeholders within the network of ASF surveillance and control. Firstly, participants were asked to name all the stakeholders they can think of that are in some way involved in the ASF network. During the process of listing the stakeholders, hunters explained, how they saw the role of each of the listed stakeholder in the control of ASF. The quantity of contacts (from no contact to daily contact) was then assessed between the listed stakeholders and hunters by using arrows (supplementary file, Fig. 2). Finding a consensus among the participants, the quantity of contacts was investigated in both directions, from the defined stakeholder to the hunter and vice versa. Subsequently, five different smileys, visualizing five different gradations of happiness (very unhappy, unhappy, neutral, happy, very happy) were given to each participant. They were asked to individually use one of the smileys to illustrate the quality of each of the contacts between the listed stakeholders and the hunters. For the semi-quantitative analysis, each of the smileys was assigned to a rank (supplementary file, Fig. 2).

Using a flow diagram, the knowledge of the hunters about the ASF surveillance system was evaluated. Participants were asked to illustrate and discuss the flow of information in case of the detection of a wild boar carcass by them. Hunters had to discuss in groups and reach consensus, which stakeholders would they contact first in case of a suspected ASF case in wild boar, what would happen with this information and which stakeholders would be involved in the further process.

Proportional piling was used to evaluate the hunters’ trust in the ASF surveillance system and its potential to effectively control ASF in wild boar. 100 glass pebbles were provided to each group of hunters. The participants were asked to form a consensus and proportionally divide the pebbles by their trust and their distrust in the effectiveness of the ASF surveillance system, respectively. Subsequently, only the pebbles that were assigned to the trust in the system were further distributed between the different stakeholders listed in the flow diagram, proportionally to the hunters’ trust in their work within the ASF surveillance system. During the implementation of the different participatory tools and the discussions, the moderator regularly probed the statements and decisions of the participants to ensure correct understanding and to stimulate further discussions.

#### 3. Part: evaluation of ASF control measures

The questionnaire study [17] conducted in Lithuania in July 2020, i.e. prior to the participatory study, revealed several ASF control measures that were determined as highly ineffective and unacceptable by the responders. These measures were presented to the participating hunters in the focus group meetings and are presented below:

1. Including additional forces like army and police (for wild boar shooting and/or carcass search).
2. Selective hunting of female wild boar.
3. Ban of supplementary feeding.
4. Ban of driven hunting.
5. Ban of individual hunting.

Within focus group discussions, hunters were firstly asked, whether they agreed with the survey results indicating that these measures were ineffective to control ASF. Subsequently, the participants discussed, what could help to improve the effectiveness and particularly the acceptability of these measures by hunters.

### Quantitative analyses

Contact frequency between hunters and other stakeholders was evaluated by assigning a specific value (from 0 to 3) to each arrow given (supplementary file, Fig. 1). The mean quantity of contact between the defined stakeholder and the hunters was calculated including the values of all groups. To evaluate the quality of the contacts, each smiley was assigned to a rank (supplementary file, Fig. 1). An average value of perceived quality was first calculated for all contacts in the individual focus groups and then for all focus groups.

For the assessment of the trust of hunters in the surveillance system and the performance of involved stakeholders, proportional piling was used. The average of pebbles assigned to trust and distrust in the system was calculated for all groups together. Subsequently, only the pebbles assigned to “trust” were further divided between the listed stakeholders in the flow diagram. Thus, the number of pebbles given to each stakeholder was standardized to 100. Therefore, the number of pebbles given to each stakeholder was divided by the number of pebbles that was assigned to “trust” and multiplied with 100. Accounting for the different number of stakeholders that were listed in the flow diagram by each group, the weighted sum of pebbles given to each stakeholder was calculated. This was done by dividing the number of stakeholders listed in each individual group by the sum of stakeholders mentioned in all groups. The result was multiplied with the amount of pebbles given to each stakeholder yielding in a comparable value for “trust” in each stakeholder [10].

## Supplementary Information

**Additional file 1: Table 1.** Semi-structured interview: Coding structure and response frequency of participatory meetings with hunters in Lithuania. **Figure 1.** Values of the average trust in the performance of stakeholders listed in the flow diagram to effectively implement ASF control measures in Lithuania. **Figure 2.** Visualization tools to define quantity and quality of the contact between hunters and other stakeholders involved in the ASF network.

## Data Availability

The data supporting the results of this study are mainly qualitative data extracted from transcripts originating from group discussions. The original data used for the analyses can be obtained from the corresponding author.
