# Supplementary material for: The perceptions of Lithuanian hunters towards African swine fever using a participatory approach
Source: BMC Vet Res. 2022 Nov 14;18:401. doi: 10.1186/s12917-022-03509-9 (PMC9660124; doi:10.1186/s12917-022-03509-9)
Supplement: Supplementary file 1 — Additional file 1: Table 1. Semi-structured interview: Coding structure and response frequency of participatory meetings with hunters in Lithuania. Figure 1. Values of the average trust in the performance of stakeholders listed in the flow diagram to effectively implement ASF control measures in Lithuania. Figure 2. Visualization tools to define quantity and quality of the contact between hunters and other stakeholders involved in the ASF network. [file 12917_2022_3509_MOESM1_ESM.docx]

Table 1. Semi-structured interview: Coding structure and response frequency of participatory meetings with hunters in Lithuania.

| **Code group** | **Code subgroup** | **No. of times mentioned** | **No. of focus groups mentioning the code** |
| --- | --- | --- | --- |
| Known ASF control measures | Ban of driven hunting | 1 | 1 |
|  | Ban of supplementary feeding | 4 | 4 |
|  | Biosecurity implementation | 1 | 1 |
|  | Effective removal of carcasses | 5 | 4 |
|  | Physical barriers | 1 | 1 |
|  | Wild boar population control | 1 | 1 |
| Consequences of ASF persistence in the Lithuanian wild boar population | Difficult work for hunters | 4 | 4 |
|  | Economic impact for pig keepers | 4 | 3 |
|  | Financial cost for hunters | 4 | 3 |
|  | Inconvenient ASF testing procedures | 4 | 2 |
|  | Lack of information for hunters regarding ASF | 2 | 2 |
|  | Reduced wild boar populations | 9 | 3 |
|  | Ruined hobby for hunters | 8 | 4 |
| Potential routes of virus introduction into the Lithuanian wild boar population | Migration of wild boar from affected countries | 6 | 6 |
|  | Pigs breeding complexes | 2 | 2 |
|  | Various fomites import from affected countries | 9 | 5 |
| Transmission pathways of the virus | Contact between wild boar | 2 | 2 |
|  | Contaminated feed | 4 | 4 |
|  | Contaminated trash, food leftovers | 3 | 3 |
|  | Dense wild boar population | 1 | 1 |
|  | Lack of biosecurity | 10 | 7 |
|  | Liquids from infected wild boar | 1 | 1 |
|  | Movement of sick animals | 11 | 5 |
|  | People movement | 12 | 7 |
|  | Wild boar carcasses | 4 | 3 |


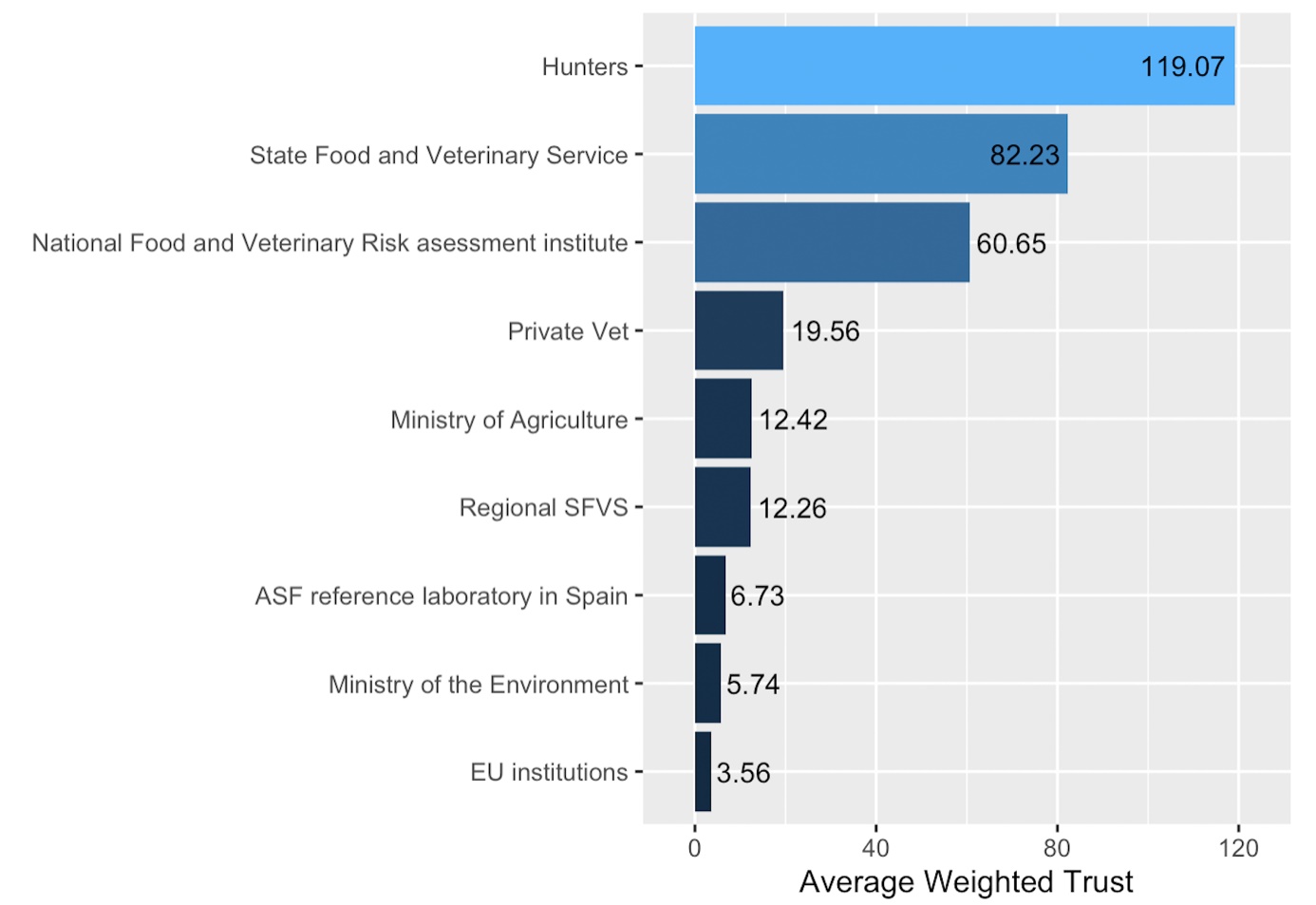


Figure 1: Values of the average trust in the performance of stakeholders listed in the flow diagram to effectively implement ASF control measures in Lithuania.


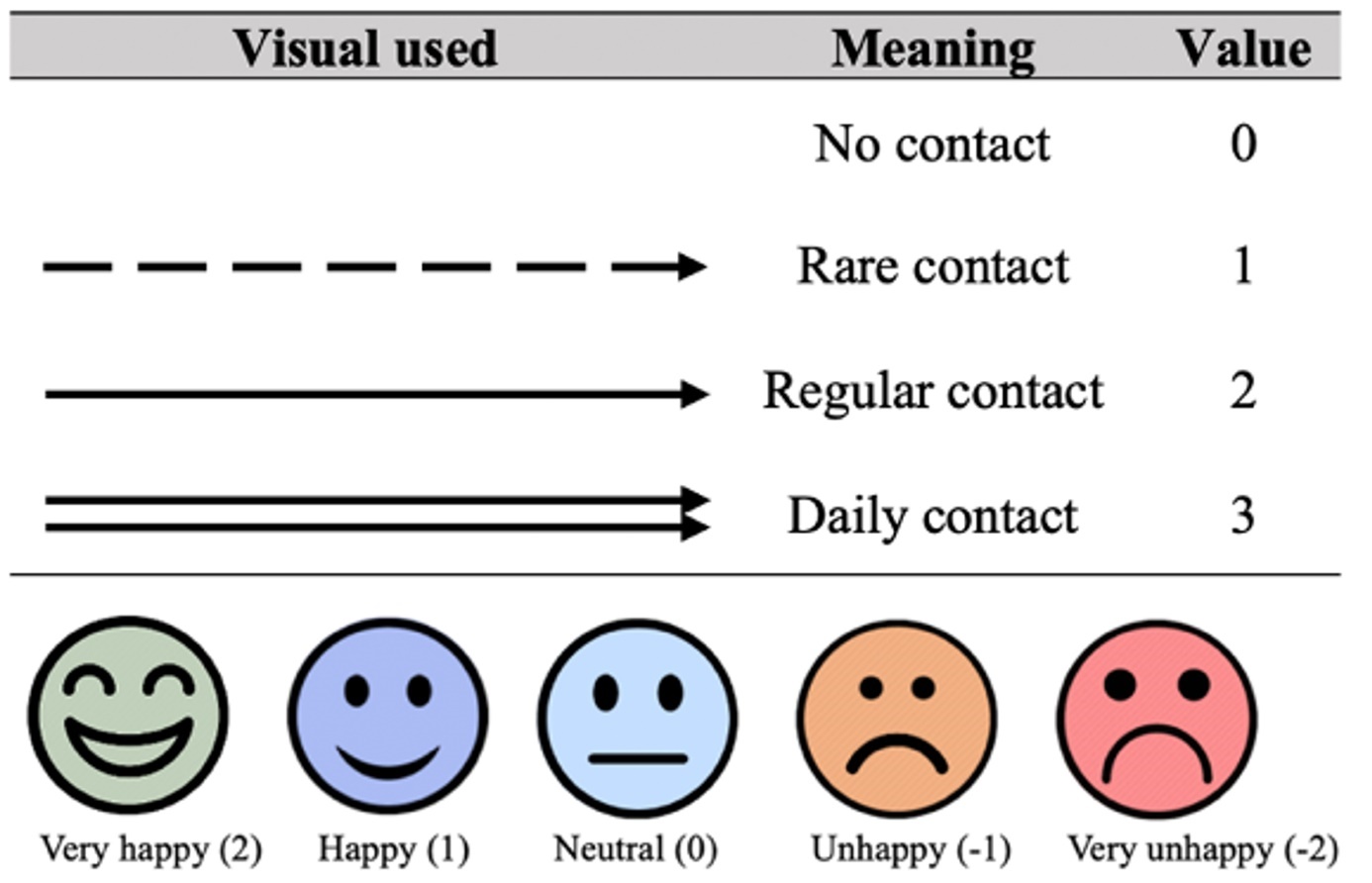


Figure 2: Visualization tools to define quantity and quality of the contact between hunters and other stakeholders involved in the ASF network.
